# Treatment of Naturally Occurring Periodontitis in Dogs With a New Bio-Absorbable Regenerative Matrix

**DOI:** 10.3389/fvets.2022.916171

**Published:** 2022-06-21

**Authors:** Jerzy Pawel Gawor, Peter Strøm, Ana Nemec

**Affiliations:** ^1^Klinika Weterynaryjna Arka, Kraków, Poland; ^2^Specialistdyrehospitalet, Holte, Denmark; ^3^Dentistry and Oral Surgery Department, Small Animal Clinic, Veterinary Faculty, University of Ljubljana, Ljubljana, Slovenia

**Keywords:** dog, periodontitis, regenerative treatment, gelatin tissue scaffold, split-mouth design

## Abstract

Although periodontal disease is one of the most common (oral) diseases in dogs, an effective treatment approach to periodontitis lacks. The aim of this study was to evaluate the safety and efficacy of a regenerative, bio-absorbable implant biomaterial made of medical-grade porcine gelatin, which is cross-linked by transglutaminase into a porous scaffold for the treatment of periodontitis in dogs in a clinical setting. Nine client-owned dogs were included in this multicenter, prospective interventional clinical study. A split-mouth design was used to treat any teeth with periodontitis; teeth on one side of the mouth were treated with open periodontal therapy alone (control teeth) and teeth on the other side were treated with open periodontal therapy and the tested implant (teeth treated with the implant). A recheck under general anesthesia was performed 3 months after the initial treatment and included periodontal probing, dental radiographs, and/or cone-beam computed tomography (CBCT) of the teeth included in the study. This revealed a reduction of the probing depth (PD) at all teeth, but in teeth treated with the implant, a statistically significant improvement (average 2.0 mm) over control teeth (average 1.0 mm) was diagnosed. Similarly, alveolar bone height was increased at most of the teeth, but in teeth treated with the implant, a statistically significant improvement (average 1.26 mm palatally and 1.51 mm buccally) over control teeth (average 0.58 mm palatally and 0.7 mm buccally) was observed for the buccal site. Open periodontal therapy alone improves clinical parameters and alveolar bone height in dogs with periodontitis, which is further significantly improved by the addition of the implant used.

## Introduction

Periodontal disease has long been considered one of the most common (oral) diseases of dogs with a significant welfare impact (1). However, an effective regenerative treatment approach to its advanced form–periodontitis–is still lacking (2–4). The hallmark of periodontitis is attachment loss, where gingiva, periodontal ligament, cementum, and bone are affected due to the chronic inflammatory process associated with the shift in the oral microbiome and several other risk factors (3). Treatment of periodontitis is currently aimed at (mostly mechanical) infection control and the creation of an environment amenable to repair or, at best, partial regeneration of the lost complex periodontal tissues (2, 4). The degree of regeneration depends on the type of the cells/tissues that first repopulate the area of lost periodontium tissues; therefore, regenerative procedures mostly involve the use of a barrier to prevent repopulation by gingival epithelial cells, which are the fastest to repopulate the healing pocket. Repopulation of the treated pocket with epithelium results in repair by a long junctional epithelium instead of regeneration with bone, cementum, and periodontal ligament (2, 4).

Treatment planning in periodontitis cases has to take into consideration several factors (client-, patient-, and environment-related), with the pattern of alveolar bone loss that plays a significant role when applying regenerative treatment techniques. Infrabony pockets (i.e., periodontal pockets with a base apical to the alveolar margin occurring in conjunction with vertical bone loss) with more bony walls surrounding them have greater potential for the regeneration of lost periodontal tissues as compared to infrabony pockets with less bony walls, and different therapeutic approaches will be applied in these cases than in the treatment of suprabony pockets (i.e., periodontal pockets with a base coronal to the alveolar margin occurring in conjunction with horizontal bone loss) (5, 6).

The classic approach to periodontal tissue regeneration is guided tissue regeneration that employs the use of a physical barrier (barrier membrane with or without a particulate graft) between the prepared root surface and the gingival tissues that mechanically prevents apical migration of the epithelium. This technique is currently indicated for the treatment of stage 2 furcation lesions and selected two- or three-walled infrabony pockets, while suprabony pockets are generally considered poor candidates for guided tissue regeneration. More recent approaches, however, are based on tissue engineering with biologic mediators that manipulate signaling molecules, scaffolds, and/or cells and the exact indications for these procedures have yet to be determined (2, 4).

The aim of this study was to evaluate the safety and efficacy of a regenerative, bio-absorbable implant biomaterial made of medical-grade porcine gelatin, which is cross-linked by transglutaminase into a porous scaffold (ReGum™, BioChange Ltd, Yokneam, Israel) (referred to in the text as “the implant”) for the treatment of periodontitis in dogs in a clinical setting. The implant (in a liquid form) has recently been successfully used for the management of urethral sphincter mechanism incompetence in client-owned dogs (7). Gelatin is produced from collagen by a denaturation process in which most of its cellular binding sites are preserved. These sites hold an important role in signal transduction cell activity regulation (8–10), promoting its ability to serve as a tissue scaffold. Its' biocompatibility, adhesiveness, mechanical properties, and low immunogenicity make gelatin an ideal biomaterial to use as an implant for tissue regeneration (11–13). The crosslinking of gelatin polymer with transglutaminase leads to the formation of a stable matrix. The porous structure of the implant presents a high surface area with an abundance of cellular binding sites, allowing migration of surrounding cells and stimulation of tissue growth (14, 15).

## Materials and Methods

This multicenter, prospective interventional clinical study included 9 client-owned dogs. Eight of the dogs had mesaticephalic heads and one was brachycephalic. All dogs were considered systemically healthy based on anamnesis, pre-anesthetic clinical examination, and results of complete blood count and blood biochemistry. To be included in the study, the dogs must not have been treated with nonsteroidal anti-inflammatory medication, corticosteroids, and/or antibiotics 3 months prior, and an informed consent form was obtained from all clients.

Initially (T0) and at the 3-month recheck (T90), all dogs were evaluated under general anesthesia following established routine and standardized protocols. The detailed oral and dental examination included full-mouth periodontal probing (using UNC 15 probe, Integra Miltex, Tuttlingen, Germany) and dental charting and was performed according to the American Veterinary Dental College (AVDC) guidelines for dogs. Clinical findings were supported by full-mouth dental radiographic examination using the size 2 and 4 imaging plates (Progeny, Midmark, USA or Sopix, Acteon, England) and/or cone-beam computed tomography (CBCT) (NewTom, 5G XL, Bologna, Italy) (cases by JG). Intraoral dental radiography was exposed in the same projections and positions both at T0 and at T90. The interpretation of dental radiographs included assessment of the alveolar bone (quality, height, and the edge at the inter-radicular and inter-dental spaces), periodontal ligament space width, and regularity. CBCT diagnostic imaging was performed at T0 and T90 that evaluated 18 teeth (9 treated with the implant and 9 control teeth), pertaining to 7 dogs. Scans were analyzed with the use of NNT viewer, software provided by the manufacturer (version: 10.1; QR SRL, Verona, Italy). At both times, the volumetric assessment of dentition was set up for the same high-resolution mode (10 × 10 cm with 0.15 mm layers). Both readings were made in the same orientations of axes and locations measuring alveolar bone height with the use of maximum magnification. To evaluate the effect of the treatments on the alveolar bone, the distance between the alveolar bone level (ABL) and cementoenamel junction (CEJ) was measured.

Regional nerve blocks were performed as clinically indicated, and all teeth were scaled supra and subgingivally with an ultrasonic scaler (iM3 42–12, Sydney, Australia or Newtron P5 BLED, Acteon, England). Teeth with perio-endo lesions, severely mobile teeth, and/or teeth with any other pathology were extracted as clinically indicated.

A split-mouth design was used to treat any teeth with advanced periodontitis (PD2 to early PD4); teeth on one side of the mouth were treated with open periodontal therapy (standard of care) (control teeth) and teeth on the other side were treated with open periodontal therapy and the implant (teeth treated with implant). Randomization was achieved by a coin toss (teeth of the right side of the mouth received the implant when the coin fell with the number up). At all the treated teeth, an initial sulcular incision was performed, followed by one or two vertical releasing incisions to enable reflection of a full-thickness mucoperiosteal flap (6) as clinically indicated. Root planing was performed with a curette (#1/2 Gracey Curette, Integra, Miltex, Tuttlingen, Germany). On the control side, the flap was replaced to its original position and sutured with poliglecaprone 25, 5–0 (Monosyn, Braun, Hessen, Germany or Monocryl, Johnson & Johnson, New Jersey, USA) in a simple interrupted manner immediately upon completion of the root planing, while at the teeth treated with an implant, the implant was placed in the defect before the flap was replaced to its original position and sutured in the same manner as on the control side. Before placement, the sterile implant was cut to size and soaked in sterile Ringer's lactate solution (Fresenius Kabi, Graz, Austria) for 30 s as per the instructions of the manufacturer, prolonging the standard procedure for about 2 min per tooth treated with the implant.

Due to the length of the procedures, all dogs received perioperative antibiotic therapy with ampicillin (Ampicillin TZF, Polfa Tarchomin, Poland or Ampicillin Stada, STAD Nordic, Denmark) 20 mg/kg IV at induction and none of the dogs received post-operative antibiotic therapy. All dogs were treated with a non-steroidal anti-inflammatory medication (meloxicam (Metacam, Boehringer Ingelheim, Germany) 0.2 mg/kg upon induction and continued with 0.1 mg/kg/24 hours perorally for 4 days). All dogs were sent home with a topical chlorhexidine gluconate of 0.12% oral rinse (Paroex, Sunstar, Etoy, Switzerland) to be used twice daily for 2 weeks. All animals were offered soft food later the same day of the procedure. Soft food was given until the first recheck 2 weeks after the procedure. To control for any difference in oral home care, the clients were instructed not to brush their dog's teeth until the recheck. Diet was not standardized, but the clients were asked not to change the diet (apart from softening it in the first 2 weeks) during the study.

A recheck under general anesthesia was performed 3 months (T90) after the initial treatment (time was determined based on what is usually recommended for treatment rechecks) and included periodontal probing, dental radiographs, and/or CBCT of the teeth, which are also included in the study. All dogs were evaluated, treated, and rechecked by the same examiner (JG 8 dogs or PS 1 dog).

For statistical evaluation, the probing depth (PD) data were analyzed for 11 pairs of teeth, and the CBCT bone measurements on the buccal and palatal/lingual side were analyzed for 9 pairs of teeth (control teeth vs. teeth treated with implant). The relatively small sample size prevented assumptions about data distribution, therefore, a non-parametric Wilcoxon signed-rank test was employed. The paired t-tests with normality assumptions were used to compare the means of the two groups. The data were analyzed using the SPSS version 28.0 program. A significant difference was considered when *p* < 0.05.

## Results

Nine dogs and 22 teeth were included in the study. The mean age of the dogs was 8 years and 11 months and the mean bodyweight was 16.3 kg. Among the treated 22 teeth, 16 teeth were three-rooted, 4 two-rooted, and 2 single-rooted (canine teeth). Two teeth were located in the mandible and 20 in the maxilla. All procedures were uneventful and all periodontal probing and CBCT measurements before (T0) and 3 months after the treatment (T90) are presented in Tables 1, 2.

**Table 1:** Clinical parameters of the treated teeth at the presentation (T0) and 3-month recheck (T90).

**Patient number**	**Tooth treated with implant**							**Control tooth**						
	**Tooth**	**Gingival bleeding**		**Probing**		**Furcation**		**Tooth**	**Gingival bleeding**		**Probing**		**Furcation**	
	**number**	**Index**		**depth [mm]**		**involvement**		**number**	**index**		**depth [mm]**		**involvement**	
		**T0**	**T90**	**T0**	**T90**	**T0**	**T90**		**T0**	**T90**	**T0**	**T90**	**T0**	**T90**
1	108	2	1	4	2	1	1	208	1	0	3	2	1	1
2	108	1	1	5	5	2	2	208	1	1	4	4	2	2
3	207	2	1	6	5	2	2	107	2	1	6	5	2	2
4	209	2	0	8	4	2	2	109	1	0	8	4	2	0
5	108	2	1	5	3	1	0	208	1	1	3	5	1	1
	109	1	2	5	3	1	1	209	1	1	4	4	1	1
6	208	2	1	5	3	3	3	108	2	1	5	4	3	3
	204	1	0	8	6	N/A	N/A	104	1	0	6	5	N/A	N/A
7	108	2	1	4	2	2	2	208	2	1	4	2	2	2
8	108	2	1	8	5	3	3	208	2	1	6	3	2	2
9	308	3	2	5	3	2	2	408	3	2	4	4	2	2

*N/A, not applicable for a single-rooted tooth*.

**Table 2:** Cone-beam computed tomography (CBCT) measurements of the distance between alveolar bone level (ABL) and cementoenamel junction (CEJ) of the treated teeth at the presentation (T0) and 3-month recheck (T90).

**Patient number**	**Tooth treated with implant**					**Control tooth**				
	**Tooth number**	**ABL-CEJ distance [mm]**				**Tooth number**	**ABL-CEJ distance [mm]**			
		**T0b**	**T90b**	**T0p**	**T90p**		**T0b**	**T90b**	**T0p**	**T90p**
1	108	5.4	3.5	3.3	2.5	208	3.7	2.6	2.6	3.1
2	108	3.9	4.0	5.3	3.8	208	5.5	5.4	4.1	3.6
4	209	5.8	3.9	1.8	0	109	6.2	5.2	2.3	1.7
5	108	3.6	2.7	1.7	1.6	208	2.2	2.2	2.4	2.0
	109	5.3	5.0	3.0	2.4	209	5.3	5.0	2.6	2.6
6	208	7.0	6.0	5.6	4.6	108	5.1	4.4	5.7	4.8
	204	9.0	7.9	9.0	7.4	104	7.6	7.5	6.9	4.6
7	108	6.8	2.6	4.1	3.4	208	7.3	5.1	5.1	4.4
8	108	5.0	2.6	8.2	5.0	208	5.3	4.5	3.0	2.7

*b, buccal, p, palatal/lingual*.

**Figure 1:**
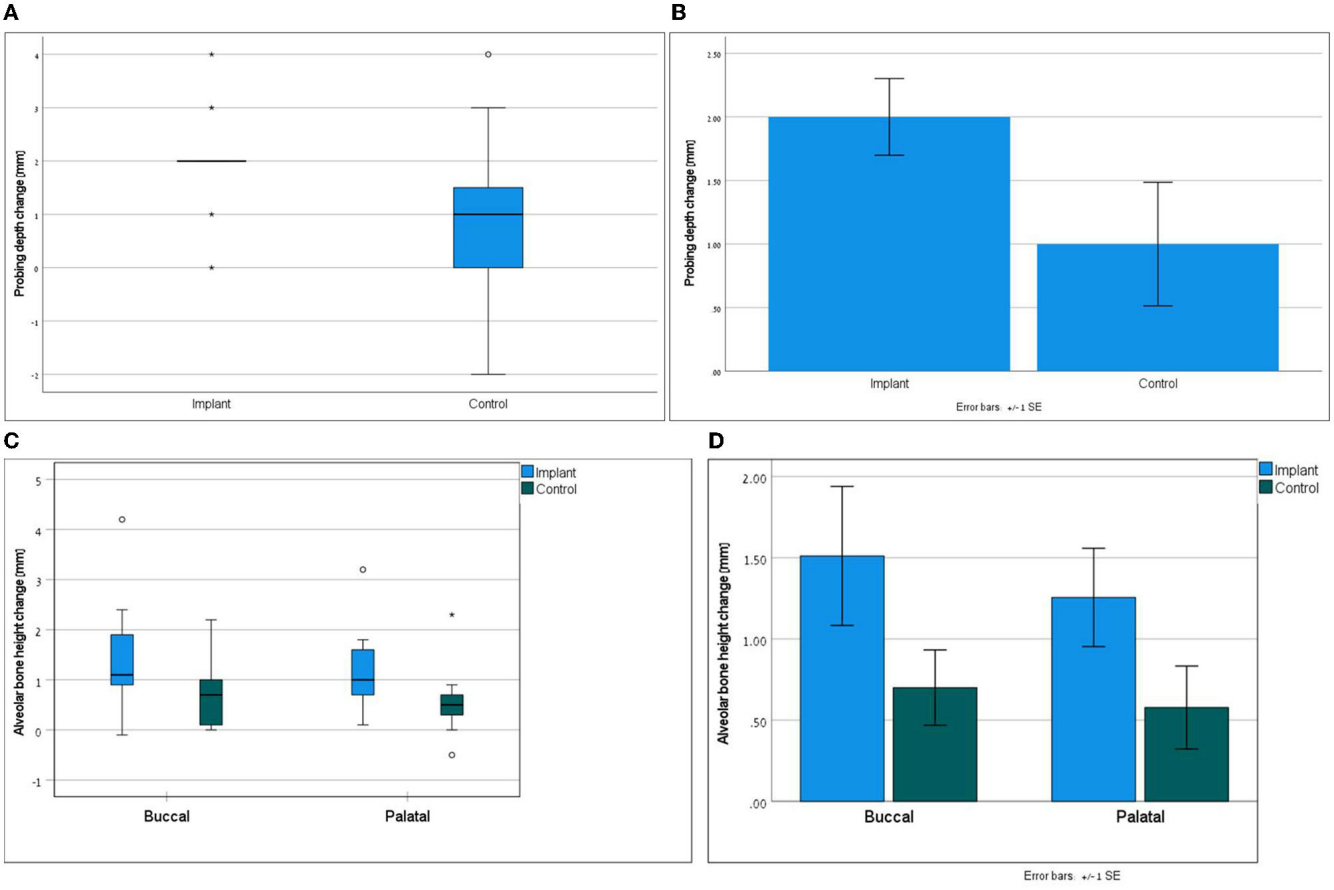
Part **(A)** demonstrates the non-parametric distribution of probing depth; Wilcoxon signed ranks test; *p* = 0.026. Part **(B)** presents the mean differences of probing depth between the groups; paired *t*-test with normality assumption; *p* = 0.024. Part **(C)** demonstrates the non-parametric distribution of alveolar bone height; Wilcoxon signed ranks test; *p* = 0.017 for buccal and *p* = 0.093 for palatal. Part **(D)** presents the mean differences in alveolar bone height between the groups; paired *t-*test with normality assumption *p* = 0.009 for buccal and *p* = 0.097 for palatal.

Clinically, the assessment of the treated teeth revealed completely healed flaps at all the teeth. At T90, gingival bleeding index (GBI) remained unchanged or was improved when compared with T0 for most of the treated teeth. PD at T90 as compared to T0 was reduced by an average of 1.0 (0–4) mm at the teeth treated with open periodontal therapy alone and by 2.0 (0–4) mm at the teeth treated with open periodontal therapy and the implant. PD at the teeth was treated with open periodontal therapy and the implant was improved significantly more as compared to the control teeth treated with open periodontal therapy alone (Figures 1A,B and Table 1). Furcation involvement mainly remained at the same stage (Table 1).

**Figure 2:**
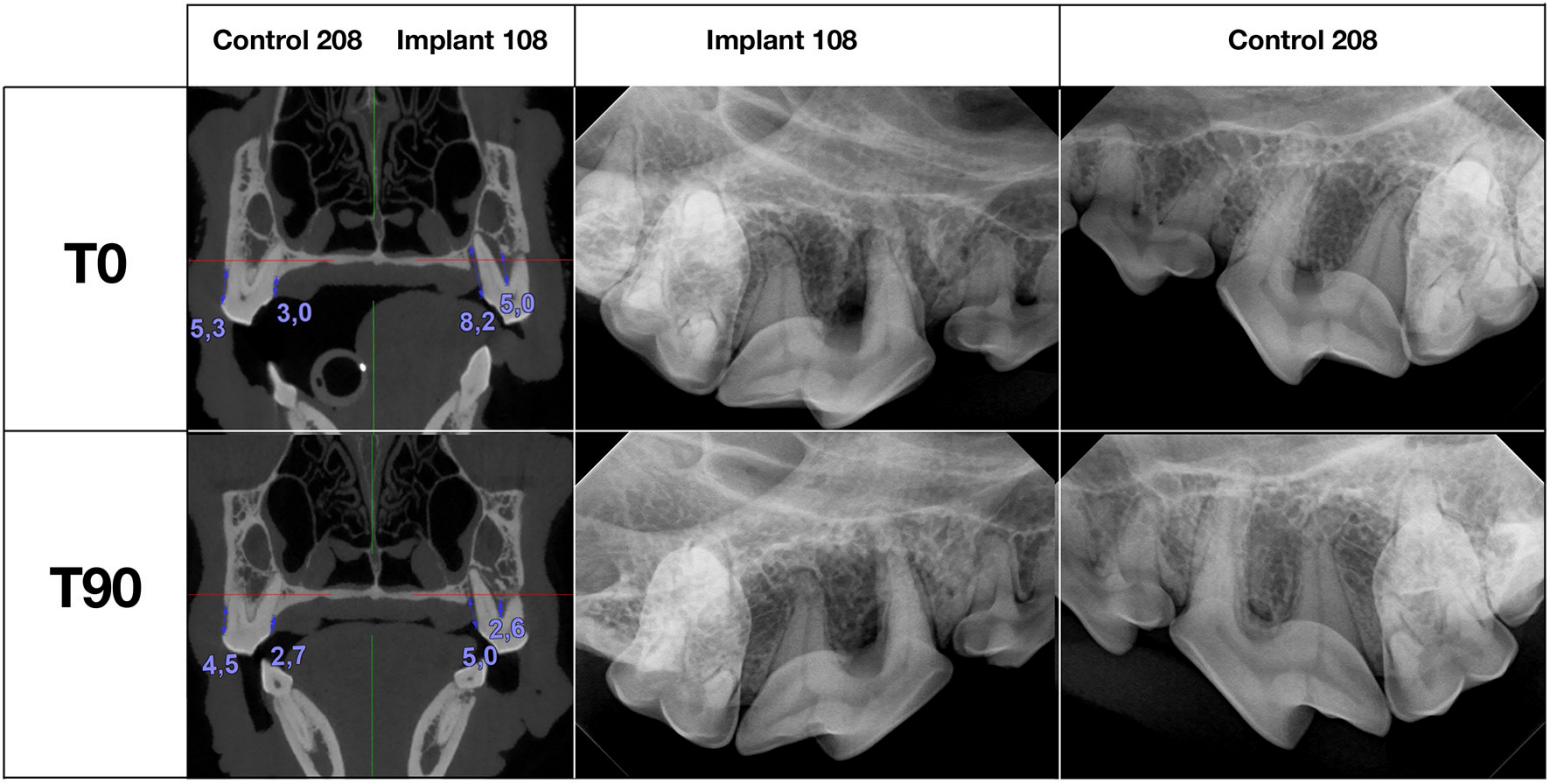
Cone-beam computed tomography (CBCT) and radiographic findings at T0 and T90 in dog 8.

Radiographically treated areas presented increased alveolar bone height at most of the teeth, which was also confirmed with CBCT in 7 dogs (Figures 1C,D, 2, and Table 2). Alveolar bone height at T90 as compared to T0 was increased by an average of 0.7 (0–0.2) mm buccally and 0.58 (−0.5–2.3) mm palatally at the teeth treated with open periodontal therapy alone and 1.51 (−0.1–4.2) mm buccally and 1.26 (0.1–3.2) mm palatally at the teeth treated with open periodontal therapy and the implant. Buccal alveolar bone height at the teeth treated with open periodontal therapy and the implant was improved significantly more as compared to the teeth treated with open periodontal therapy alone (Figures 1C,D, 2, and Table 2).

## Discussion

The results of this study show that open periodontal therapy alone improves selected clinical parameters and bone height at 3 months post-treatment in dogs with naturally occurring periodontitis. These parameters can be further significantly improved by the addition of the regenerative, bio-absorbable implant biomaterial made of medical-grade porcine gelatin used in our study. Importantly, the implant used in our study is also safe; no side effects of the treatment were observed immediately or 3 months after the treatment in any of the dogs included in this study. The implant also proved to be easy to handle in a clinical setting (flexible one component implant with a long shelf-life that enables final shaping at the point of care), and the procedures were not significantly prolonged as compared to open periodontal therapy alone.

Most of the research in periodontal tissue regeneration is experimental/pre-clinical and only rarely dogs with naturally occurring periodontitis are included (2). Studies using experimentally induced periodontitis are generally well-controlled with detailed clinical, imaging, histological, and even molecular evaluation of the outcome of the treatments and hence reveal promising approaches for periodontal regeneration (16–21). However, experimentally induced periodontitis may represent different microbial and inflammatory dynamics as compared to naturally occurring diseases, and therefore, limitations to directly extrapolate the data to the clinical setting need to be considered.

On the other hand, several techniques using a combination of bone graft and membranes (22, 23) have been described to successfully treat advanced naturally occurring periodontitis in dogs as indicated by marked PD reduction and radiographically evident alveolar bone gain, but the lack of control treatment/comparison to (open) periodontal therapy alone limits the interpretation about the amount of the efficacy of the material(s) alone.

In a very recent study similar to ours by Kornsuthisopon et al. (24), laboratory-housed dogs with pre-existing periodontitis (PD 3–5 mm) were included in the split-mouth design study evaluating the efficacy of the treatment with autologous platelet-rich fibrin (PRF) combined with open periodontal therapy in comparison to open periodontal therapy alone. Similar to our study, the authors found a trend (statistically non-significant) of decreased PD in teeth treated with open periodontal therapy alone, while the addition of PRF resulted in a significant improvement of PD 14, 21, and 56 days after the treatment. Similar to our findings, a trend of decreased GBI was observed for all the teeth. However, the radiographic analysis revealed no significant impact of PRF treatment on the alveolar bone gain at 56 days, which is contrary to our findings, but the follow-up time in our study was longer (90 days). In general, veterinary studies with longer follow-up times are indicated to better evaluate the long-term benefits of surgical periodontal procedures (25).

Similarly, improved periodontal indices (i.e., gingivitis index, gingival crevicular fluid, PD, and attachment loss) 6 and 12 weeks post-periodontal therapy were also found in one of the first clinical trials using a split-mouth design, where the authors reported that the improvement was significantly better if a doxycycline polymer filling was used in addition to closed periodontal therapy alone (26). The reported mean reduction in PD 3 months post-treatment was 1.04 mm at the teeth receiving doxycycline polymer and 0.62 mm on the control side (26), which is about half as much improvement as observed in our study. Another randomized controlled clinical study evaluated the effect of a clindamycin hydrochloride gel as an addition to closed periodontal therapy to treat periodontitis in two groups of client-owned dogs (27). The authors reported significant clinical benefit (i.e., improved PD, GBI) of clindamycin hydrochloride gel 3 months after the treatment, with clindamycin-treated dogs showing a mean reduction in PD of 0.7 mm, when compared with 0.1 mm in control dogs (27). Contrary to Zetner and Rothmueller's and Johnston et al. studies, a more recent randomized, blinded, controlled clinical study found no clinically relevant benefit of adding either doxycycline or clindamycin gel application to closed periodontal therapy 12 weeks after the treatment. The authors still confirmed that all three types of the treatments result in improvement of periodontal indices (i.e., GBI, plaque and calculus index, and PD), which was the greatest for the most severely affected teeth (PD 5–5.5 mm) (28). Ideally, however, pockets deeper than 4–5 mm would be approached by an open periodontal therapy to allow visualization of the defect for an appropriate treatment (6). With dogs assigned to different treatment groups, it is also impossible to control for inter-individual response to the treatment, which is better addressed in studies using a split-mouth design. Additionally, none of these studies evaluated alveolar bone gain. In summary, the implant used in our study results in better periodontitis control (as measured by PD and alveolar bone gain) at 3 months post-treatment as compared to locally delivered either doxycycline or clindamycin gels and also eliminates the use of an antibiotic. While local and/or systemic use of antibiotics may be indicated in the treatment of specific cases of periodontitis, antibiotic use should ideally be avoided to minimize the risk of bacterial resistance to anti-microbials (29). Moreover, infectious agents other than bacteria are likely involved in the development and progression of periodontitis (30) as are other factors (e.g., nutrition, stress, chewing habits, oral care, host response, genetic factors, and age) (3).

The two biggest limitations of our study are the lack of histological examination of the treated areas (24) and no baseline control (without treatment), but such criteria would be unethical in a clinical setting. Despite marked improvement in clinical and imaging indices of periodontitis, without histological analysis, it remains unclear if the implant used in this study truly enabled the regeneration of periodontal tissues or the implant use resulted just in an enhanced repair (2). However, even if the use of the implant used in our study does not result in regeneration, its use relieves the symptoms, improves periodontal health to a greater extent than open periodontal therapy alone, and can therefore be recommended in the treatment of naturally occurring periodontitis in dogs. Moreover, even better results could be expected, if oral home care with daily tooth brushing (31) was instituted once the soft tissues healed, although for the easier control in this study oral home care was omitted.

## Conclusion

Based on the data of this study, open periodontal therapy alone improves clinical parameters and alveolar bone height in dogs with periodontitis, which is further significantly improved by the addition of the regenerative, bio-absorbable implant biomaterial made of medical-grade porcine gelatin as used in our study. Therefore, the use of this implant can be recommended in addition to open periodontal therapy in dogs with advanced periodontitis, considering the careful selection of the patients and teeth that will truly benefit from the treatment. This implant is not only effective and safe but also easy to handle and with a long shelf-life. Further studies that include larger cohorts of clinical patients with longer follow-up times are encouraged to better understand the clinical performance of the implant long term.

## Data Availability Statement

The original contributions presented in the study are included in the article/supplementary material, further inquiries can be directed to the corresponding author.

## Ethics Statement

Ethical review and approval was not required for the animal study because this was a clinical study using a regenerative material, that was previously used in a clinical setting for different disease. The study included client-owned dogs and all owners agreed to the treatment, that was standard-of-care, with the addition of the regenerative material. Written informed consent was obtained from the owners for the participation of their animals in this study.

## Author Contributions

All authors listed have made a substantial, direct, and intellectual contribution to the work and approved it for publication.

## Funding

This work was supported by BioChange Ltd, Yokneam, Israel.

## Conflict of Interest

The authors declare that this study received funding from BioChange Ltd, Yokneam, Israel. The funder had the following involvement in the study: material design and development and the the decision to submit this article for publication.

## Publisher's Note

All claims expressed in this article are solely those of the authors and do not necessarily represent those of their affiliated organizations, or those of the publisher, the editors and the reviewers. Any product that may be evaluated in this article, or claim that may be made by its manufacturer, is not guaranteed or endorsed by the publisher.

## References

[1] Summers JF O'Neill DG Church D Collins L Sargan D Brodbelt DC. Health-related welfare prioritisation of canine disorders using electronic health records in primary care practice in the UK. BMC Vet Res. (2019) 15:163. 10.1186/s12917-019-1902-0 31118035 PMC6532203

[2] Ward E. A review of tissue engineering for periodontal tissue regeneration. J Vet Dent. (2022) 39:49–62. 10.1177/08987564211065137 34935526

[3] Wallis C Holcombe LJ. A review of the frequency and impact of periodontal disease in dogs. J Small Anim Pract. (2020) 61:529–40. 10.1111/jsap.13218 32955734

[4] Fiani N Lommer MJ. Reparative and regenerative periodontal surgery. In: Verstraete FJM Lommer MJ Arzi B editors. Oral and Maxillofacial Surgery in Dogs and Cats. 2nd ed. Elsevier, St. Louis Missouri (2020). p. 202–10.

[5] Hale FA Gorrel CE. Principles of periodontal surgery. In: Verstraete FJM Lommer MJ Arzi B editors. Oral and Maxillofacial Surgery in Dogs and Cats. 2nd ed. Elsevier, St. Louis Missouri (2020). p. 180–5.

[6] Stepaniuk KS Staley BB. Periodontal flaps and mucogingival surgery. In: Verstraete FJM Lommer MJ Arzi B editors. Oral and Maxillofacial Surgery in Dogs and Cats. 2nd ed. Elsevier, St. Louis Missouri (2020). p 194–201.

[7] Chen H Shipov A Segev G. Evaluation of cross-linked gelatin as a bulking agent for the management of urinary sphincter mechanism incompetence in female dogs. J Vet Intern Med. (2020) 34:1914–9. 10.1111/jvim.15857 32686187 PMC7517489

[8] Kuwahara K Yang Z Slack GC Nimni ME Han B. Cell delivery using an injectable and adhesive transglutaminase–gelatin gel. Tissue Eng Part C Methods. (2010) 16:609–18. 10.1089/ten.tec.2009.0406 19757996

[9] Ito A Mase A Takizawa Y Shinkai M Honda H Hata K. Transglutaminase-mediated gelatin matrices incorporating cell adhesion factors as a biomaterial for tissue engineering. J Biosci Bioeng. (2003) 95:196–9. 10.1016/S1389-1723(03)80129-9 16233392

[10] Ivaska J Heino J. Adhesion receptors and cell invasion: mechanisms of integrin-guided degradation of extracellular matrix. Cell Mol Life Sci. (2000) 57:16–24. 10.1007/s000180050496 10949578 PMC11146885

[11] Gupta D Santoso JW McCain ML. Characterization of gelatin hydrogels cross-linked with microbial transglutaminase as engineered skeletal muscle substrates. Bioengineering. (2021) 8:6. 10.3390/bioengineering8010006 33418892 PMC7825108

[12] Lu T Yu K Kuo S Cheng N Chuang E. Yu J. Enzyme-crosslinked gelatin hydrogel with adipose-derived stem cell spheroid facilitating wound repair in the murine burn model. Polymers. (2020) 12:2997. 10.3390/polym12122997 33339100 PMC7765510

[13] Yamamoto S Hirata A Ishikawa S Ohta K Nakamura K Okinami S. Feasibility of using gelatin-microbial transglutaminase complex to repair experimental retinal detachment in rabbit eyes. Graefes Arch Clin Exp Ophthalmol. (2013) 251:1109–14. 10.1007/s00417-012-2245-8 23283484

[14] Chen P Yang K Wu C Yu J Lin F Sun J. Fabrication of large perfusable macroporous cell-laden hydrogel scaffolds using microbial transglutaminase. Acta Biomater. (2014) 10:912–20. 10.1016/j.actbio.2013.11.009 24262131

[15] Yung CW Wu LQ Tullman JA Payne GF Bentley WE Barbari TA. Transglutaminase crosslinked gelatin as a tissue engineering scaffold. J Biomed Mater Res A. (2007) 83:1039–46. 10.1002/jbm.a.31431 17584898

[16] Shirakata Y Imafuji T Nakamura T Kawakami Y Shinohara Y Noguchi K. Periodontal wound healing/regeneration of two-wall intrabony defects following reconstructive surgery with cross-linked hyaluronic acid-gel with or without a collagen matrix: a preclinical study in dogs. Quintessence Int. (2021) 308–16. 10.3290/j.qi.b937003 33533237

[17] Imber J Bosshardt DD Stähli A Saulacic N Deschner J Sculean A. Pre-clinical evaluation of the effect of a volume-stable collagen matrix on periodontal regeneration in two-wall intrabony defects. J Clin Periodontol. (2021) 48:560–9. 10.1111/jcpe.13426 33471389

[18] Huang R Tai W Ho M Chang P. Combination of a biomolecule-aided biphasic cryogel scaffold with a barrier membrane adhering PDGF-encapsulated nanofibers to promote periodontal regeneration. J Periodontal Res. (2020) 55:529–38. 10.1111/jre.12740 32096217

[19] Wei L Teng F Deng L Liu G Luan M Jiang J. Periodontal regeneration using bone morphogenetic protein 2 incorporated biomimetic calcium phosphate in conjunction with barrier membrane: a pre-clinical study in dogs. J Clin Periodontol. (2019) 46:1254–63. 10.1111/jcpe.13195 31518453 PMC6899729

[20] Antunes AA Grossi-Oliveira GA Martins-Neto EC Gonçalves De Almeida AL Salata LA. Treatment of circumferential defects with osseoconductive xenografts of different porosities: a histological, histometric, resonance frequency analysis, and micro-CT study in dogs. Clin Implant Dent Relat Res. (2015) 17(Suppl. 1):e202–20. 10.1111/cid.12181 24283568

[21] Suaid FF Ribeiro FV Rodrigues TL Silvério KG Carvalho MD Nociti FH Jr. Autologous periodontal ligament cells in the treatment of class II furcation defects: a study in dogs. J Clin Periodontol. (2011) 38:491–8. 10.1111/j.1600-051X.2011.01715.x 21392047

[22] Alterman JB Huff JF. Guided tissue regeneration in four teeth using a liquid polymer membrane. J Vet Dent. (2016) 33:185–94. 10.1177/0898756416676564 28327066

[23] Stepaniuk KS Gingerich W. Evaluation of an osseous allograft membrane for guided tissue regeneration in the dog. J Vet Dent. (2015) 32:226–32. 10.1177/089875641503200403 27012060

[24] Kornsuthisopon C Pirarat N Osathanon T Kalpravidh C. Autologous platelet-rich fibrin stimulates canine periodontal regeneration. Sci Rep. (2020) 10:1850. 10.1038/s41598-020-58732-x 32024893 PMC7002419

[25] Cortellini P Cortellini S Bonaccini D Tonetti MS. Modified minimally invasive surgical technique in human intrabony defects with or without regenerative materials. J Clin Periodontol. (2022) 49:528–36. 10.1111/jcpe.13627 35415940

[26] Zetner K Rothmueller G. Treatment of periodontal pockets with doxycycline in beagles. Vet Ther. (2002) 3:441–52. 12584682

[27] Johnston TP Mondal P Pal D MacGee S Stromberg AJ Alur H. Canine periodontal disease control using a clindamycin hydrochloride gel. J Vet Dent. (2011) 28:224–9. 10.1177/089875641102800402 22416621

[28] Martel DP Fox PR Lamb KE Carmichael DT. Comparison of closed root planing with versus without concurrent doxycycline hyclate or clindamycin hydrochloride gel application for the treatment of periodontal disease in dogs. J Am Vet Med Assoc. (2019) 254:373–9. 10.2460/javma.254.3.373 30668243

[29] Sarkiala EM. Use of antibiotics and antiseptics. In: Verstraete FJM Lommer MJ Arzi B editors. Oral and Maxillofacial Surgery in Dogs and Cats. 2nd ed. Elsevier, St. Louis Missouri (2020). p 14–21.

[30] Niemiec BA Gawor J Tang S Prem A Krumbeck JA. The mycobiome of the oral cavity in healthy dogs and dogs with periodontal disease. Am J Vet Res. (2021) 83:42–9. 10.2460/ajvr.20.11.0200 34727047

[31] Allan RM Adams VJ Johnston NW. Prospective randomised blinded clinical trial assessing effectiveness of three dental plaque control methods in dogs. J Small Anim Pract. (2019) 60:212–7. 10.1111/jsap.12964 30575038

